# Machine Learning-Assisted Protein Redesign and Codon Optimization for Soluble Expression of Human Lactoferrin in *Escherichia coli*

**DOI:** 10.3390/foods15213913

**Published:** 2026-10-31

**Authors:** Jun Feng, Huijia Yan, Shuzhen Cheng, Zhenyu Wang, Ming Du

**Affiliations:** 1 SKL of Marine Food Processing & Safety Control, School of Food Science and Technology, Dalian Polytechnic University, Dalian 116034, China; fengjun_dlpu@163.com (J.F.); y18742013250@163.com (H.Y.); chengshuzhen0547@yeah.net (S.C.); 2 National Engineering Research Center of Seafood, Collaborative Innovation Centre of Seafood Deep Processing, Dalian Polytechnic University, Dalian 116034, China; 3 College of Food Science and Engineering, Dalian Ocean University, Dalian 116023, China

**Keywords:** human lactoferrin, codon optimization, protein engineering, soluble expression

## Abstract

Recombinant lactoferrin (LF) production in *Escherichia coli* (*E. coli*) is limited by poor solubility and inclusion body formation. To address this, a computational-experimental workflow was established, combining protein redesign and structural screening with a deep learning-based codon optimization strategy. Candidate mutants were screened using SurfSol and structurally assessed using AlphaFold 3. Subsequently, a bidirectional gated recurrent unit (BiGRU) model was benchmarked against five algorithms. In the test-subset comparison, the BiGRU model achieved the highest area under the receiver operating characteristic curve (ROC-AUC) and average precision (AP) values, recording a ROC-AUC of 0.777 and an AP of 0.720. Qualitative Western blot screening detected soluble-fraction anti-His-reactive bands for ELF2 among the tested constructs in *E. coli*. In lipopolysaccharide (LPS)-stimulated NCM460 cells, treatment with the ELF2-derived Ni-NTA-enriched preparation was associated with lower interleukin-6 (IL-6) and tumor necrosis factor-α (TNF-α) levels and higher interleukin-10 (IL-10) levels. Attribution of these cytokine changes to the redesigned LF requires protein identification and purity assessment. Nevertheless, the qualitative expression screen supports ELF2 as a promising candidate for further characterization.

## 1. Introduction

Lactoferrin (LF), an iron-binding glycoprotein, exhibits extensive antimicrobial, anti-inflammatory, and immunomodulatory properties, making it of considerable interest for applications in infant nutrition, functional foods, and biopharmaceuticals [1,2]. At present, most commercially available LF is derived from natural milk sources, but this route is often limited by low production efficiency, high purification cost, and batch-to-batch variation [3]. Among different LF homologs, human lactoferrin (hLF) is of particular interest because of its close relevance to human physiology and its potential value in infant formula and food-related health applications [4,5]. However, obtaining hLF from natural sources remains particularly challenging because human milk is limited in availability and subject to ethical and logistical constraints [6]. Recombinant production therefore represents an attractive alternative for the scalable supply of hLF [7,8]. *Escherichia coli* (*E. coli*) is a prevalent expression platform due to its fast growth, straightforward genetics, and cost-effective cultivation [9,10]. *Escherichia coli* Nissle 1917 (EcN) is a well-characterized probiotic strain that has increasingly been explored as a microbial chassis for the production and delivery of heterologous biomolecules [11]. Recent work has further developed EcN-based recombinant protein expression systems; for example, a low-temperature inducible EcN platform supported the production of both sfGFP and thermophilic trehalose synthase, demonstrating its utility for heterologous protein production [12]. Nevertheless, soluble expression of hLF in *E. coli* is still difficult. As a eukaryotic protein, hLF is prone to misfolding and aggregation in the bacterial cytoplasm, often leading to the formation of inclusion bodies and reduced soluble yield [9]. In addition, codon usage bias can influence translation kinetics and co-translational folding, further limiting efficient soluble expression [13]. Thus, enhancing hLF production in *E. coli* may require coordinated optimization of both protein and coding-sequence levels, beyond mere codon replacement [14]. Recent host-engineering studies further support the feasibility of recombinant hLF production for food-related applications [15].

At the protein level, computational protein design provides an opportunity to improve intrinsic sequence properties such as solubility while maintaining structural integrity and function. Integrating sequence redesign with solubility prediction and structural evaluation can therefore facilitate the identification of protein variants that are more suitable for heterologous expression before coding-sequence optimization [16]. Consistent with this rationale, rational modification of the iron-binding site of hLF has been reported to enhance recombinant expression while retaining experimentally evaluated protein function [17]. Traditional codon optimization strategies at the coding-sequence level primarily depend on static statistical metrics like the codon adaptation index (CAI). These metrics summarize codon usage preferences but fail to fully capture the interaction between coding sequences and the host’s translation machinery. In practice, these methods often focus on synonymous codon frequencies while overlooking local sequence context and its potential effects on translation efficiency and protein folding [18]. As a result, improved codon adaptation does not always translate into better soluble expression. Deep learning has recently provided a more flexible framework for modeling translational behavior because sequence-based models can capture contextual dependencies that are difficult to represent using conventional codon statistics alone [19]. For example, multilayer perceptrons (MLPs) can effectively process numerical feature inputs but do not explicitly model the ordered structure of coding sequences. Recurrent architectures such as bidirectional gated recurrent unit (BiGRU) networks can process codon sequences bidirectionally, providing a framework for representing codon-order and sequence-context information along the coding sequence [20]. Transformer-based models [21] can represent long-range interactions through self-attention, yet their computational cost increases substantially with sequence length, which may limit their efficiency in iterative sequence design and large-scale optimization settings.

Gated recurrent units (GRUs) offer a practical alternative for classification based on coding-sequence-derived features. With a relatively simple gating architecture, GRU-based models have been widely applied to capture informative dependencies in sequential data and have also shown utility in food-related prediction tasks, including bioactive peptide screening and food quality classification [22,23,24,25]. Such characteristics make GRU-based models potentially suitable for learning relationships between coding-sequence-derived patterns and heterologous expression outcomes [26].

In this study, we developed an integrated protein redesign and codon optimization pipeline that combines protein-level solubility screening and structural assessment with bidirectional gated recurrent unit (BiGRU)-based coding-sequence prediction. Candidate hLF variants were first screened according to predicted solubility and structural similarity, after which synonymous coding sequences of the selected variant were optimized using the trained model. The optimized hLF constructs were experimentally evaluated in *E. coli* to determine their effects on soluble protein expression. Furthermore, cellular responses to the ELF2-derived preparation were evaluated in lipopolysaccharide (LPS)-stimulated NCM460 cells by measuring the inflammatory mediators interleukin-6 (IL-6), tumor necrosis factor-α (TNF-α), and interleukin-10 (IL-10). Together, these analyses provide a combined computational and experimental framework for prioritizing hLF constructs with favorable soluble-expression profiles and examining cellular responses to the recovered preparation, and may offer a useful strategy for recombinant food bioactive protein development.

## 2. Materials and Methods

### 2.1. Materials, Strains, Plasmids, and Reagents

*Escherichia coli *Nissle 1917 (EcN; T0147, Miaoling Biotechnology Co., Ltd., Wuhan, China), originally obtained commercially and subsequently maintained in our laboratory, was used as the host strain for recombinant lactoferrin expression. The plasmid pGFPmut3.1 was purchased from Shanghai Haiji Haoge Biotechnology Co., Ltd. (Shanghai, China) and used as the cloning vector. The human colonic epithelial cell line NCM460 was purchased from Shanghai Zhong Qiao Xin Zhou Biotechnology Co., Ltd. (Shanghai, China) and used for the cell assays. Restriction enzymes, DNA polymerase, ligase, ampicillin, and other routine molecular biology reagents were purchased from Sangon Biotech (Shanghai, China). Lipopolysaccharide (LPS) and isopropyl β-D-1-thiogalactopyranoside (IPTG) were purchased from Beijing Solarbio Science & Technology Co., Ltd. (Beijing, China). The anti-His tag antibody was purchased from MCE (Monmouth Junction, NJ, USA), and the enhanced chemiluminescence (ECL) detection reagent was obtained from Beyotime Biotechnology (Shanghai, China). Dulbecco’s modified Eagle’s medium (DMEM), fetal bovine serum (FBS), and penicillin–streptomycin were purchased from Gibco (Thermo Fisher Scientific, Waltham, MA, USA). Enzyme-linked immunosorbent assay (ELISA) kits for interleukin-6 (IL-6), tumor necrosis factor-α (TNF-α), and interleukin-10 (IL-10) were purchased from Shanghai Enzyme-linked Biotechnology Co., Ltd. (Shanghai, China).

### 2.2. Sequence Retrieval, Protein Redesign, and Computational Screening

The amino acid sequence of hLF was retrieved from the UniProt database (Entry: P02788, TRFL_HUMAN). Based on UniProt annotation and reported structural and functional information on human lactoferrin, residues 1–278 and 336–619 were kept fixed during ProteinMPNN redesign. These regions encompass key iron-binding residues in the N- and C-lobes, including Asp60, Tyr92, Tyr192, and His253 in the N-lobe and Asp395, Tyr433, Tyr526, and His595 in the C-lobe [27,28]. ProteinMPNN redesign was performed on the remaining regions [16,29]. ProteinMPNN was run for the single-chain hLF structure using the default model (v_48_020), with a sampling temperature of 0.1 and backbone noise of 0.00 Å; other parameters were retained at their default settings.

A total of 1000 candidate variants were generated and used as the initial library for downstream screening. The predicted solubility of the candidate variants was evaluated using SurfSol, a multimodal deep-learning framework integrating protein surface, sequence, and structural information for protein solubility prediction [30]. SurfSol was run using the default settings, and the candidates were ranked according to their predicted solubility scores. The five highest-ranked variants were subjected to structural prediction using AlphaFold 3 [31] with the default server settings. Available predicted structures were subsequently compared with wild-type hLF (WT-LF) in PyMOL (version 2.6.0), and structural similarity was assessed using root-mean-square deviation (RMSD) and template modeling score (TM-score). The candidate showing a favorable combination of predicted solubility and structural similarity to WT-LF was selected as the template for subsequent synonymous codon optimization and designated Mut-LF. For the final selected variant, molecular surface properties were further analyzed using BIOVIA Discovery Studio. Hydrophobicity and ionizability surface maps were generated for both Mut-LF and WT-LF to compare their surface physicochemical characteristics. The hydrophobicity maps were used to visualize the distribution of hydrophilic and hydrophobic regions, whereas the ionizability maps were used to visualize the distribution of basic and acidic surface regions.

### 2.3. Construction of the Codon Optimization Dataset

**Figure 1:**
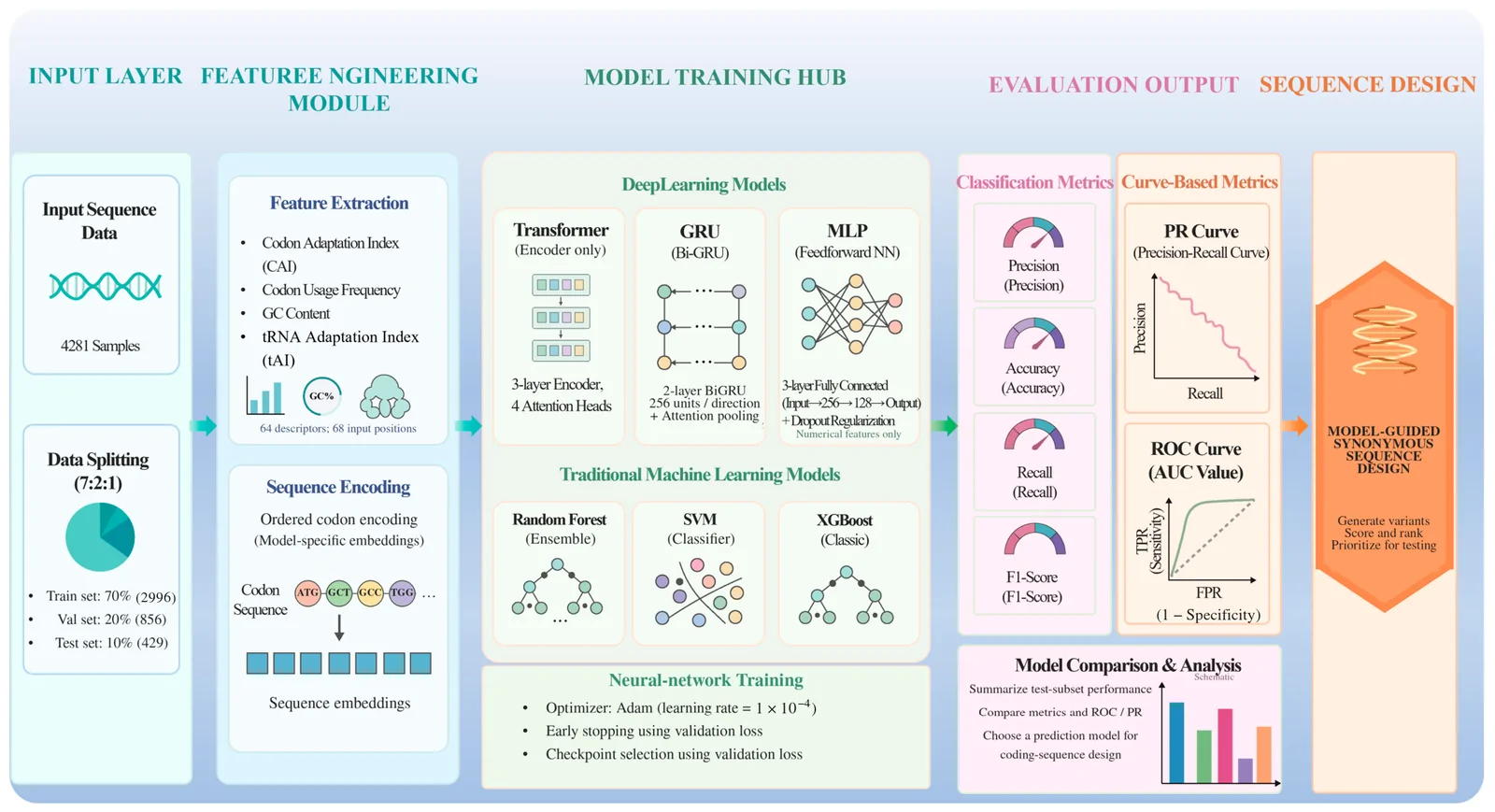
Workflow of machine learning-assisted codon optimization for human lactoferrin, including data preprocessing, model development and evaluation, and model selection for codon optimization. Network diagrams, curves, and charts are schematic.

The overall workflow of dataset construction, model development, and coding-sequence optimization is shown in Figure 1. To develop a codon optimization model for *E. coli*, a codon–expression dataset was obtained from the publicly available dataset reported by Ding et al. [32]. The original data were reprocessed and curated in-house to standardize coding-sequence annotation, expression labels, and feature representation. Exact duplicate coding sequences were removed before dataset partitioning, resulting in 4281 unique coding sequences. The corresponding protein sequences were obtained by translation of the curated coding sequences. Pairwise full-length protein-sequence identity was assessed by global sequence alignment, and sequence pairs with ≥70% identity were examined for their distribution across the training, validation, and test subsets.

The 4281 unique coding sequences were randomly divided into training, validation, and test sets at an approximate ratio of 7:2:1, corresponding to 2996, 856, and 429 samples, respectively. The training set was used for model fitting, the validation set for early stopping and checkpoint selection during neural-network training, and the test subsets for the reported model comparisons.

### 2.4. Feature Extraction

To characterize coding-sequence features relevant to heterologous expression in *E. coli*, a total of 64 sequence-level numerical features were extracted from each coding sequence, comprising codon frequencies for the 61 sense codons, tRNA adaptation index (tAI) [33], codon adaptation index (CAI) [34], and GC content. The 61 codon-frequency features were arranged in a consistent codon order across all samples.

For tAI calculation, each coding sequence was parsed into consecutive codons, and the corresponding codon weights were assigned according to the *E. coli* tAI weight table (ecoli_tai_s). The tAI value was calculated as the geometric mean of codon weights using Equation (1):

(1) $$\mathrm{tAI}=\exp\left(\left(\frac{1}{L}\right)\sum_{i=1}^{L}\ln\left(s_{i}\right)\right)$$

where *L* is the total number of codons in the coding sequence, and *s_i_* is the tAI weight assigned to the i-th codon.

For CAI calculation, codons were matched to the *E. coli* CAI weight table (ecoli_cai_weights), and the CAI value was calculated using Equation (2):

(2) $$\mathrm{CAI}=\exp\left(\left(\frac{1}{L}\right)\sum_{i=1}^{L}\ln\left(w_{i}\right)\right)$$

where *w_i_* is the relative adaptiveness weight of the *i*-th codon.

Codon-usage features were represented by the frequencies of the 61 sense codons. For each coding sequence, the frequency of codon i was calculated using Equation (3):

(3) $$f_{i}=\frac{n_{i}}{\sum_{j=1}^{61}n_{j}}$$

where n*_i_* denotes the number of occurrences of codon *i* in the coding sequence. The resulting 61 codon-frequency values were arranged in a fixed codon order and used as numerical input features.

GC content was calculated using Equation (4):

(4) $$\mathrm{GCcontent}\;\left(\%\right)=\frac{\left(G+C\right)}{\left(A+T+G+C\right)}\times 100$$

where (A), (T), (G), and (C) represent the numbers of the corresponding nucleotides in the coding sequence. The resulting 64 numerical features were normalized before model training to reduce scale-related differences among descriptors.

### 2.5. Model Architectures and Training

The curated dataset was divided into training (70%), validation (20%), and test (10%) sets. Six classification models were developed and compared for prediction of high-expression coding sequences, including Transformer, BiGRU, MLP, Random Forest, support vector machine (SVM), and XGBoost.

The Transformer model consisted of three encoder layers with four attention heads. The BiGRU model used two complementary input branches: an ordered codon-sequence branch and a numerical-feature branch. For the sequence branch, each coding sequence was parsed into consecutive codons in the 5′–3′ direction and converted into an ordered sequence of codon IDs. The 61 sense codons were assigned unique integer IDs, with 0 reserved for padding. Codon sequences were padded with zeros or truncated to a fixed length of 1000 codons, resulting in a sequence input tensor of shape [batch size, 1000]. Each codon ID was mapped to a 21-dimensional embedding vector, and the embedded sequence was processed by two bidirectional GRU layers with a hidden dimension of 256 per direction, followed by attention pooling. In parallel, the numerical-feature branch received a 68-position input containing the 64 normalized sequence-level features described in Section 2.4, comprising 61 codon-frequency features, GC content, CAI, and tAI, with four trailing zeros. These numerical features were processed separately, and the resulting representation was combined with the sequence representation from the BiGRU branch for classification. The MLP model consisted of three fully connected layers with an input–256–128–1 architecture and dropout regularization. For the traditional machine-learning models, Random Forest, SVM, and XGBoost were fitted to the numerical features.

Model selection was based on the six-model test-subset comparison, with precision used as the primary selection criterion because the downstream application involved prioritizing a limited number of high-confidence positive candidates for experimental testing. ROC-AUC and average precision (AP) were used as complementary measures of overall classification performance. Validation loss was used separately for early stopping and checkpoint selection during neural-network training.

### 2.6. Model Evaluation

Model performance on the test subsets was evaluated using accuracy, precision, recall, F1-score, area under the receiver operating characteristic curve (ROC-AUC), average precision (AP), and confusion matrices. For the confusion-matrix analysis, a probability threshold of 0.5 was used for binary classification, with predicted probabilities > 0.5 assigned to the positive class and those ≤0.5 assigned to the negative class. Accuracy was calculated using Equation (5):

(5) $$\mathrm{Accuracy}=\frac{\mathrm{TP}+\mathrm{TN}}{\mathrm{TP}+\mathrm{TN}+\mathrm{FP}+\mathrm{FN}}$$

Precision was calculated using Equation (6):

(6) $$\mathrm{Precision}=\frac{\mathrm{TP}}{\mathrm{TP}+\mathrm{FP}}$$

Recall was calculated using Equation (7):

(7) $$\mathrm{Recall}=\frac{\mathrm{TP}}{\mathrm{TP}+\mathrm{FN}}$$

The F1-score was calculated using Equation (8):

(8) $$F1=\frac{2\times \mathrm{Precision}\times \mathrm{Recall}}{\mathrm{Precision}+\mathrm{Recall}}$$

where TP, TN, FP, and FN denote true positives, true negatives, false positives, and false negatives, respectively. ROC-AUC and AP were used as threshold-independent summary metrics to assess model discrimination. All evaluation metrics were calculated using scikit-learn (version 0.24.2) [35].

### 2.7. Generation of Optimized Coding Sequences

Based on the selected redesigned lactoferrin variant, candidate coding sequences were generated by random synonymous substitution at approximately 50% of the codon positions while preserving the encoded amino acid sequence. Approximately 20,000 synonymous coding sequences were generated to increase sequence diversity. The generated sequences were screened and ranked using the trained BiGRU expression prediction model, and sequences with a predicted high-expression probability of at least 0.5 were retained.

Among the retained candidates, sequences were ranked according to their predicted high-expression probabilities. The three highest-ranking coding sequences were selected for gene synthesis and subsequent experimental screening and were designated ELF1, ELF2, and ELF3.

### 2.8. Plasmid Construction and Transformation

The coding sequences of ELF1, ELF2, and ELF3 were commercially synthesized by GENEWIZ (Azenta Life Sciences, Suzhou, China). CO-LF was generated based on the WT-LF amino acid sequence using the conventional *E. coli* codon-optimization strategy provided by GENEWIZ. The pGFPmut3.1 vector was linearized by PCR, and the CO-LF and ELF1–ELF3 coding sequences were inserted into the linearized vector by homologous recombination. A C-terminal 6×His tag was fused to each target protein for subsequent detection and purification. The primers used for plasmid construction are listed in Table S1. The resulting recombinant plasmids were confirmed by Sanger sequencing and subsequently introduced into competent EcN cells by heat-shock transformation. Transformants were selected on LB agar containing 100 μg/mL ampicillin, and confirmed recombinant clones were used for subsequent protein expression experiments.

### 2.9. Recombinant Protein Expression

Confirmed recombinant EcN clones harboring the corresponding plasmids were inoculated into 5 mL LB medium containing 100 μg/mL ampicillin and cultured overnight at 37 °C with shaking at 200 rpm. The overnight cultures were diluted 1:100 into fresh LB medium supplemented with 100 μg/mL ampicillin and grown at 37 °C until the optical density at 600 nm (OD_600_) reached 0.4–0.6.

Protein expression was then induced by adding IPTG to a final concentration of 0.5 mM, and the cultures were further incubated at 16 °C for 16 h with shaking at 200 rpm. After induction, the cells were harvested by centrifugation at 8000× *g* for 10 min at 4 °C and used for soluble protein extraction.

### 2.10. Growth Curve Analysis

To evaluate whether recombinant lactoferrin expression affected the growth of EcN, growth curves of the recombinant strains were monitored by measuring the optical density at 600 nm (OD_600_). Overnight cultures harboring the corresponding recombinant plasmids were diluted 1:100 into fresh LB medium containing 100 μg/mL ampicillin and cultured at 37 °C with shaking at 200 rpm. When the OD_600_ reached 0.4–0.6, IPTG was added to a final concentration of 0.5 mM to induce recombinant protein expression. The cultures were then continuously incubated at 37 °C with shaking at 200 rpm, and OD_600_ values were recorded at regular intervals over a 24 h cultivation period. EcN carrying the empty vector was used as the control. Growth curves were generated by plotting OD_600_ against cultivation time. All measurements were performed using three independent biological replicates.

### 2.11. Soluble Protein Extraction and Western Blot Analysis

The harvested bacterial pellets were resuspended in phosphate-buffered saline (PBS, pH 7.4) and lysed by sonication on ice. Sonication was performed using pulse cycles of 3 s on and 5 s off for a total sonication time of 15 min to avoid overheating. The lysates were centrifuged at 12,000× *g* for 20 min at 4 °C, and the supernatants were collected as the soluble protein fractions.

Total protein concentrations of the soluble fractions were determined using the bicinchoninic acid (BCA) assay. Equal amounts of total soluble protein were mixed with 5× SDS loading buffer and denatured at 100 °C for 10 min. The samples were separated by 12% sodium dodecyl sulfate–polyacrylamide gel electrophoresis (SDS–PAGE) and transferred onto polyvinylidene difluoride (PVDF) membranes. The membranes were blocked with 5% (*w*/*v*) bovine serum albumin (BSA) in Tris-buffered saline containing Tween 20 (TBST) at room temperature for 1.5 h and incubated with an anti-His tag primary antibody diluted 1:1000 overnight at 4 °C. After washing with TBST, the membranes were incubated with the corresponding horseradish peroxidase (HRP)-conjugated secondary antibody at room temperature for 1 h. The immunoreactive bands were visualized using the ECL detection system according to the manufacturer’s instructions.

The soluble expression profiles of CO-LF and ELF1–ELF3 were compared qualitatively based on the corresponding anti-His-reactive band patterns after loading equal amounts of total soluble protein. ELF2, which exhibited a detectable anti-His-reactive signal, was selected for subsequent Ni-NTA enrichment and cell-based evaluation.

### 2.12. Ni-NTA Enrichment of the ELF2-Derived Preparation

The soluble fraction of EcN lysates expressing ELF2, which encodes C-terminally 6×His-tagged Mut-LF, was enriched using nickel–nitrilotriacetic acid (Ni-NTA) agarose (QIAGEN, Hilden, Germany) under native conditions. The soluble fraction was loaded onto Ni-NTA resin equilibrated with 50 mM NaH_2_PO_4_, 300 mM NaCl, and 10 mM imidazole (pH 8.0). After washing with buffer containing 20 mM imidazole, the bound protein was eluted with 250 mM imidazole [36]. The eluted fractions were pooled, dialyzed against PBS (pH 7.4) at 4 °C, and analyzed by SDS–PAGE. The pooled, dialyzed eluate, referred to as the ELF2-derived preparation, was used in the cell assays. Total protein concentration was determined using the BCA assay.

### 2.13. Cell Viability and Anti-Inflammatory Activity Assay

Normal human colonic epithelial NCM460 cells were cultured in DMEM supplemented with 10% FBS and 1% penicillin–streptomycin at 37 °C in a humidified atmosphere containing 5% CO_2_. Cells were passaged every 2–3 days using 0.25% trypsin–ethylenediaminetetraacetic acid (EDTA) when they reached approximately 80–90% confluence.

To assess cellular responses to the test materials and determine a suitable concentration for subsequent experiments, NCM460 cells were seeded into 96-well plates at a density of 5 × 10^3^ cells/well and allowed to adhere overnight. Cells were then treated with different concentrations of commercial WT-LF or the ELF2-derived preparation for 24 h. For the preparation, doses were based on total protein measured by BCA. Cell viability was determined using the Cell Counting Kit-8 (CCK-8) assay. Briefly, 10 μL of CCK-8 reagent was added to each well, followed by incubation at 37 °C for 2 h. Absorbance was measured at 450 nm using a microplate reader, and cell viability was calculated relative to the untreated control group.

For establishment of the inflammatory model, NCM460 cells were stimulated with 1 μg/mL LPS for 12 h [37]. After LPS stimulation, cells were washed twice with PBS and then treated with commercial WT-LF or the ELF2-derived preparation at the specified concentration for 24 h. Control groups included untreated cells and LPS-treated cells without lactoferrin, serving as the inflammatory model. For comparative analysis, treatment groups included commercial wild-type human lactoferrin as a functional reference (WT-LF; L4894, Sigma-Aldrich, St. Louis, MO, USA) and the ELF2-derived preparation described in Section 2.12. Post-treatment, cell culture supernatants were centrifuged at 1000× *g* for 10 min to eliminate cellular debris. IL-6, TNF-α, and IL-10 concentrations in the culture supernatants were measured using human ELISA kits (Mlbio, Shanghai Enzyme-linked Biotechnology Co., Ltd., Shanghai, China; IL-6, cat. no. ml058097; TNF-α, cat. no. ml077385; IL-10, cat. no. ml064299). Samples from three independent biological experiments were diluted 1:1 with sample diluent and analyzed separately. For each assay, 50 μL of diluted sample or standard was incubated with 50 μL of detection antibody at 37 °C for 1 h. After three washes, 80 μL of streptavidin-HRP was added and incubated at 37 °C for 30 min, followed by washing, color development, and measurement at 450 nm. Cytokine concentrations were determined from the corresponding standard curves. The assay ranges were 3.125–200, 15.625–1000, and 0.781–50 pg/mL for IL-6, TNF-α, and IL-10, respectively, with corresponding sensitivities of 1.56, 7.81, and 0.39 pg/mL. Standard curves were generated by linear regression using the absorbance values and known concentrations of the corresponding cytokine standards, and cytokine concentrations in the samples were calculated from the resulting regression equations without normalization to viable cell number or cellular protein.

### 2.14. Statistical Analysis

Results are expressed as mean ± standard deviation (SD) from three independent biological experiments. Statistical analyses were conducted using Origin 2021 (OriginLab Corporation, USA) and SPSS 26.0 (IBM Corp., USA). Multiple-group comparisons were conducted using one-way analysis of variance (ANOVA) with Tukey’s post hoc test. Different lowercase letters in the figures indicate statistically significant differences among groups, where applicable. Statistical significance was defined as *p* < 0.05.

## 3. Results and Discussion

### 3.1. Structural Screening and Selection of the Redesigned Lactoferrin Variant

To improve the soluble expression of recombinant lactoferrin while preserving its structural integrity, protein redesign was first performed using ProteinMPNN. Residues 1–278 and 335–619 were kept unchanged during redesign based on the predefined functional/conserved-region constraints, whereas the remaining regions were redesigned to generate 1000 candidate sequences. Candidate variants were ranked according to their predicted solubility using SurfSol, followed by AlphaFold 3-based structural prediction and structural comparison with WT-LF in PyMOL.

**Figure 2:**
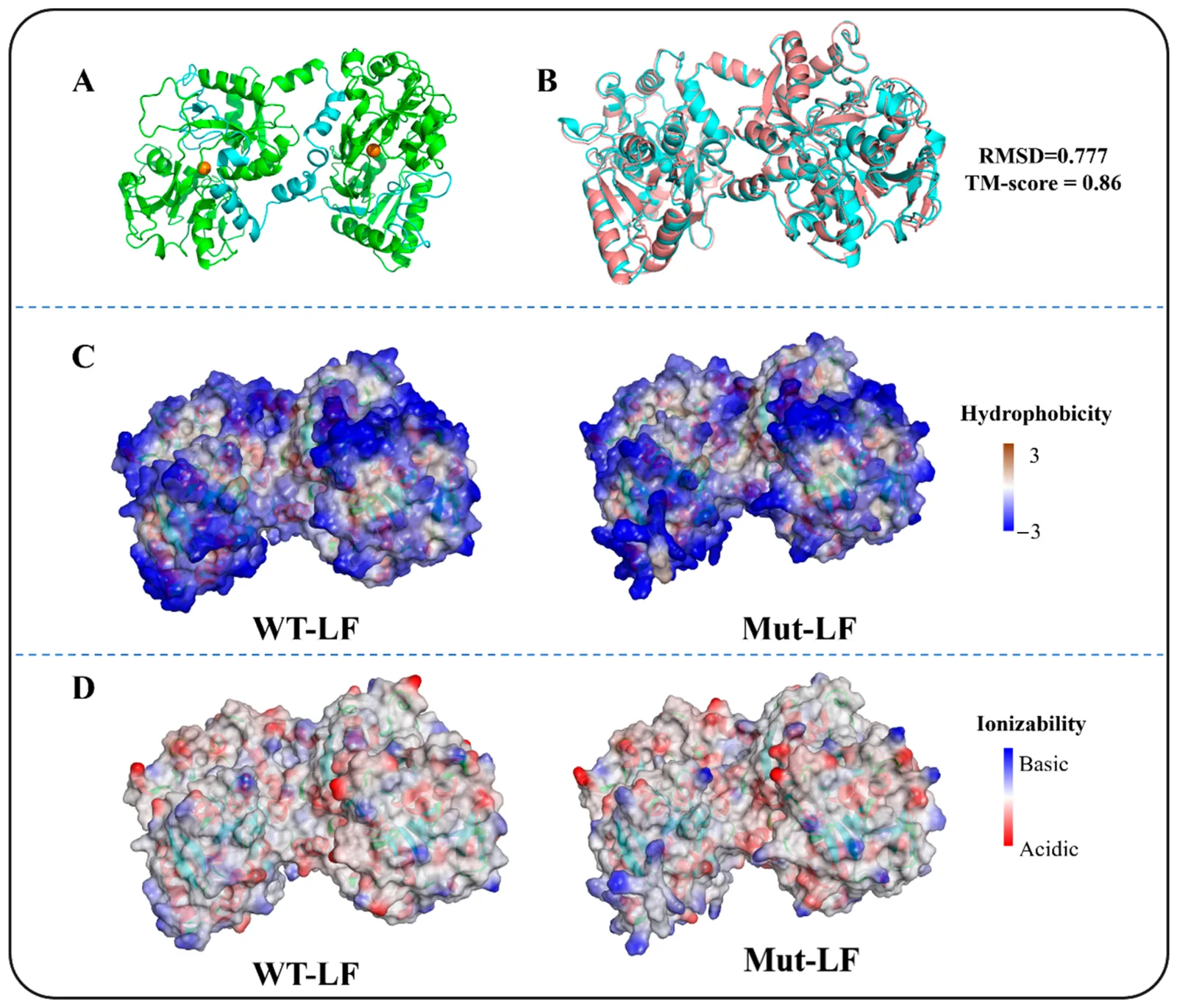
Structural comparison and surface physicochemical characterization of WT-LF and Mut-LF. (**A**) Predicted three-dimensional structure of wild-type lactoferrin (WT-LF). (**B**) Structural superposition of WT-LF and Mut-LF, showing a root-mean-square deviation (RMSD) of 0.777 Å and a TM-score of 0.86. (**C**) Surface hydrophobicity distributions of WT-LF and Mut-LF. (**D**) Surface ionizability distributions of WT-LF and Mut-LF.

The available screening results are summarized in Table 1. Mut31 showed a SurfSol-predicted solubility score of 0.3604 and, among candidates with an RMSD below 0.8 Å for which solubility data were available, had the highest predicted solubility. Figure 2A shows the predicted three-dimensional structure of WT-LF, whereas Figure 2B shows the structural superposition of WT-LF and Mut31, which revealed high predicted structural similarity with an RMSD of 0.777 Å and a TM-score of 0.86. Mut31 was therefore selected as Mut-LF for subsequent synonymous coding-sequence generation and experimental validation [38,39]. The complete amino acid sequence alignment of WT-LF and Mut31 (Mut-LF), with the substituted residues indicated, is provided in Figure S1.

To further evaluate the potential influence of protein redesign on surface physicochemical characteristics, hydrophobicity and ionizability maps were generated using BIOVIA Discovery Studio (Figure 2C,D). Qualitative comparison of the surface maps showed broadly similar patterns of hydrophobic and hydrophilic regions between WT-LF and Mut-LF, with localized differences around the redesigned regions. The ionizability maps likewise showed generally similar spatial patterns of acidic and basic surface regions. Taken together, Mut-LF combined favorable predicted solubility with high structural similarity to WT-LF and was therefore carried forward for subsequent synonymous coding-sequence generation and experimental validation. Structural similarity was used as a screening criterion rather than as direct evidence of preserved lactoferrin function. Similar structure-guided redesign strategies have been applied to other proteins using ProteinMPNN, with computationally generated variants subsequently evaluated experimentally for the desired protein properties [16,29]. These surface maps were used for qualitative comparison; quantitative surface descriptors, such as solvent-accessible surface area (SASA) or residue-level charge-distribution metrics, were not calculated in the present study.

### 3.2. Performance Comparison of Codon Expression Prediction Models

After removal of exact duplicate coding sequences, the curated dataset contained 4281 unique coding sequences. Translation of these sequences yielded 4281 distinct full-length protein sequences. Pairwise full-length sequence analysis identified only two protein pairs with ≥70% identity in the complete dataset, with a maximum identity of 72.4%; both pairs were located within the training set. No protein pair with ≥70% identity was shared across the training, validation, and test subsets, and no pair in the complete dataset reached 80% identity.

The six models were compared on their test subsets. Given the downstream objective of prioritizing a limited number of high-confidence candidates for experimental screening, greater emphasis was placed on precision and the control of false-positive predictions, while ROC-AUC and AP were considered as complementary measures of overall classification performance. As this comparison also informed model selection, the observed performance may be subject to selection-induced optimism, and independent external validation will be needed to assess the generalizability of the selected model.

**Figure 3:**
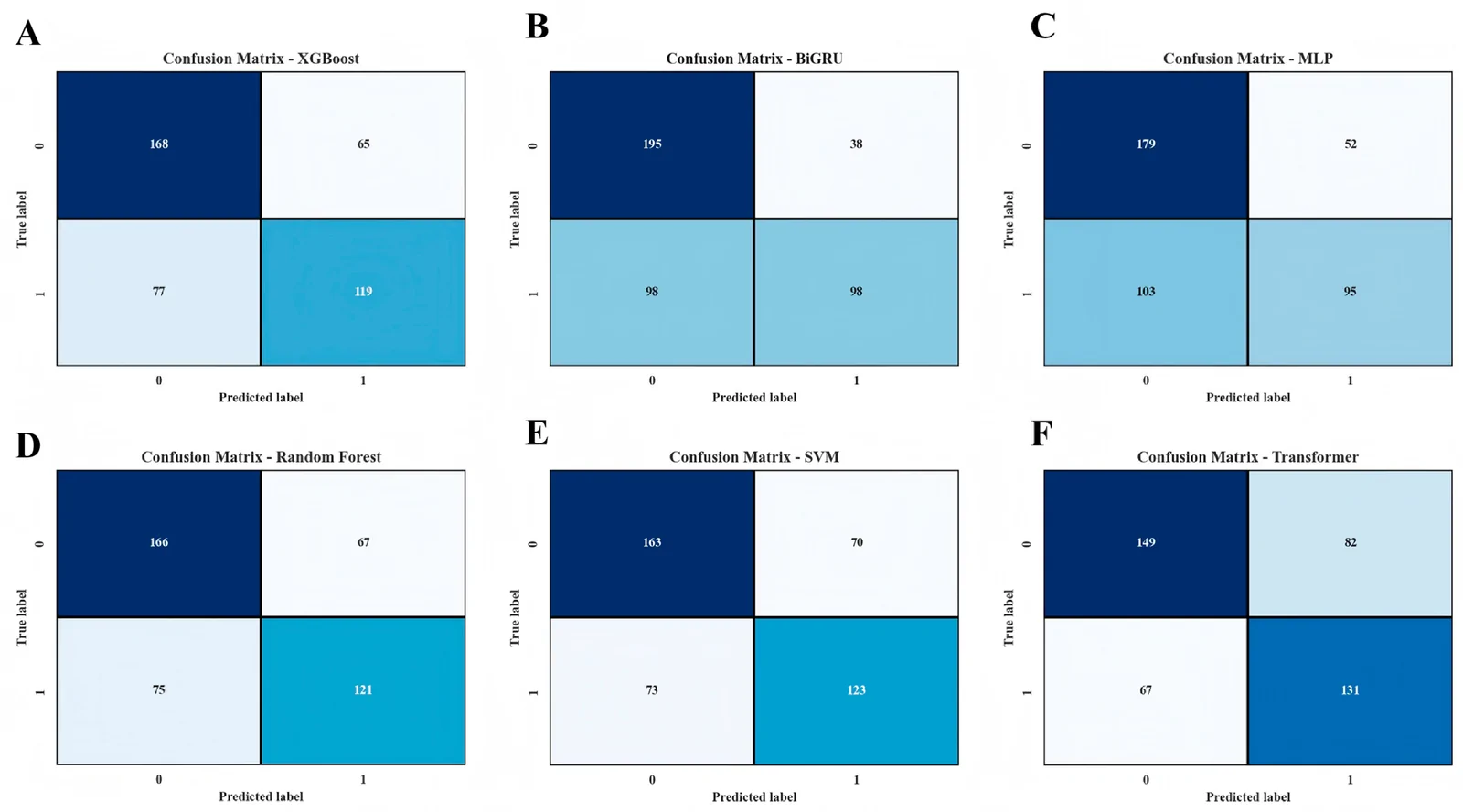
Confusion matrices of the six models for high-expression sequence prediction. Confusion matrices of (**A**) XGBoost, (**B**) BiGRU, (**C**) MLP, (**D**) Random Forest, (**E**) SVM, and (**F**) Transformer on their respective test subsets. Rows represent the true class labels and columns represent the predicted class labels; class 0 and class 1 denote low- and high-expression sequences, respectively.

The performance of the six models was examined on their test subsets using confusion matrices (Figure 3). Among the six models, BiGRU showed the lowest number of false-positive predictions, with 38 high-expression candidates incorrectly classified from the negative class. The corresponding false-positive counts for MLP, XGBoost, Random Forest, SVM, and Transformer were 52, 65, 67, 70, and 82, respectively. In this comparison, BiGRU exhibited a relatively conservative classification profile, with fewer false-positive high-expression predictions than the other models.

Although BiGRU showed a higher number of false negatives than several other models, its lower false-positive rate is relevant to the downstream screening setting, in which the number of candidate sequences that can be synthesized and experimentally evaluated is limited. In this context, reducing false-positive predictions is particularly important because each incorrectly prioritized candidate increases experimental cost and workload [32,40]. By contrast, models with higher recall identified more positive samples but also introduced a greater number of false positives.

**Figure 4:**
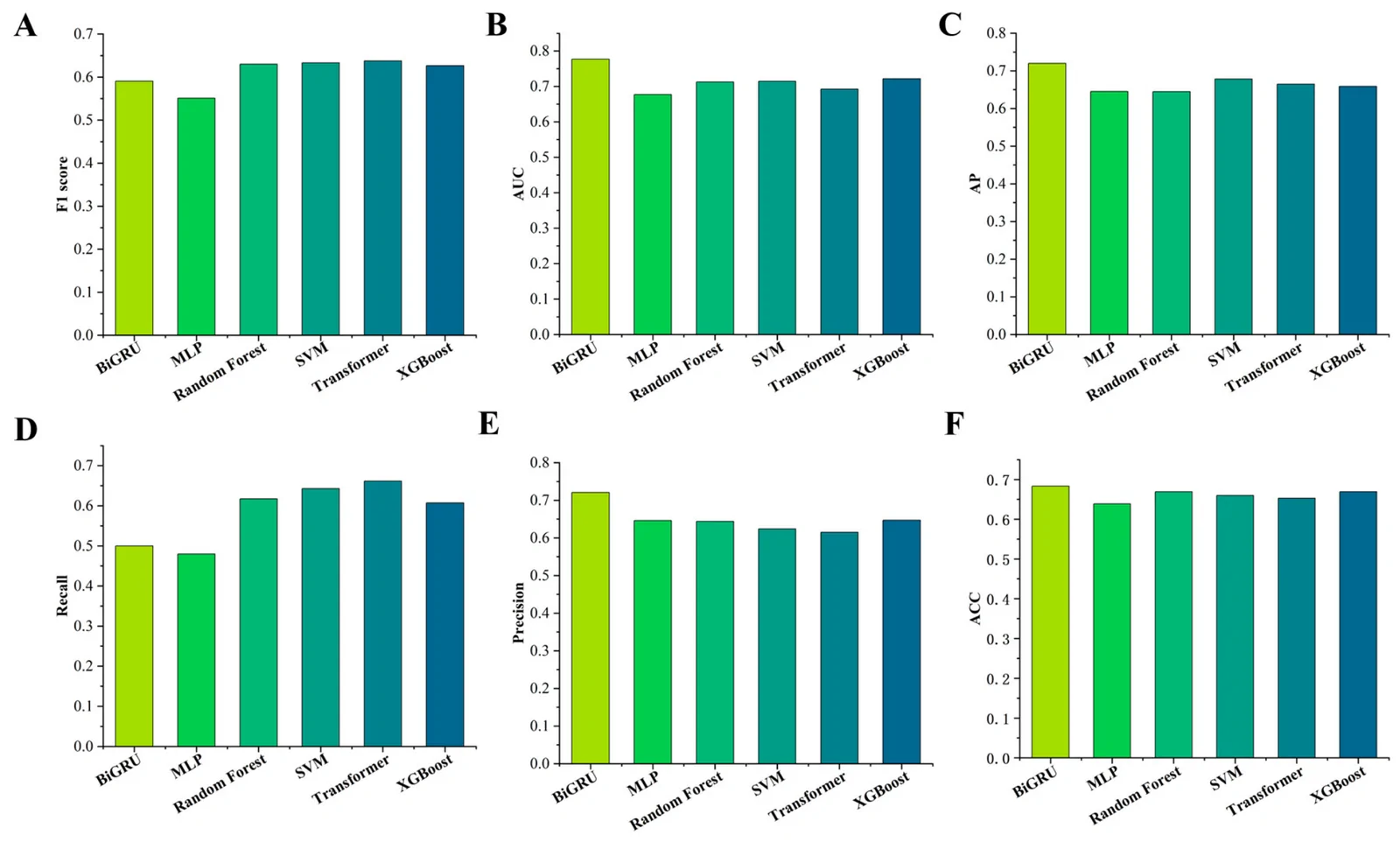
Comparative performance of six models for codon expression prediction. Performance of BiGRU, MLP, Random Forest, SVM, Transformer, and XGBoost was evaluated using six metrics: (**A**) F1-score, **(B**) area under the receiver operating characteristic curve (ROC-AUC), (**C**) average precision (AP), (**D**) recall, (**E**) precision, and (**F**) accuracy (ACC).

The overall predictive performance on the test subsets was further evaluated using F1-score, ROC-AUC, average precision (AP), recall, precision, and accuracy (Figure 4). BiGRU showed the highest observed ROC-AUC and AP values among the six models on the test subsets and also exhibited comparatively high precision and accuracy. Transformer showed the highest recall, while BiGRU had higher precision, indicating different trade-offs between these metrics. These differences were further examined across classification thresholds using ROC and precision–recall curves.

### 3.3. ROC and Precision–Recall Analysis and Model Selection

**Figure 5:**
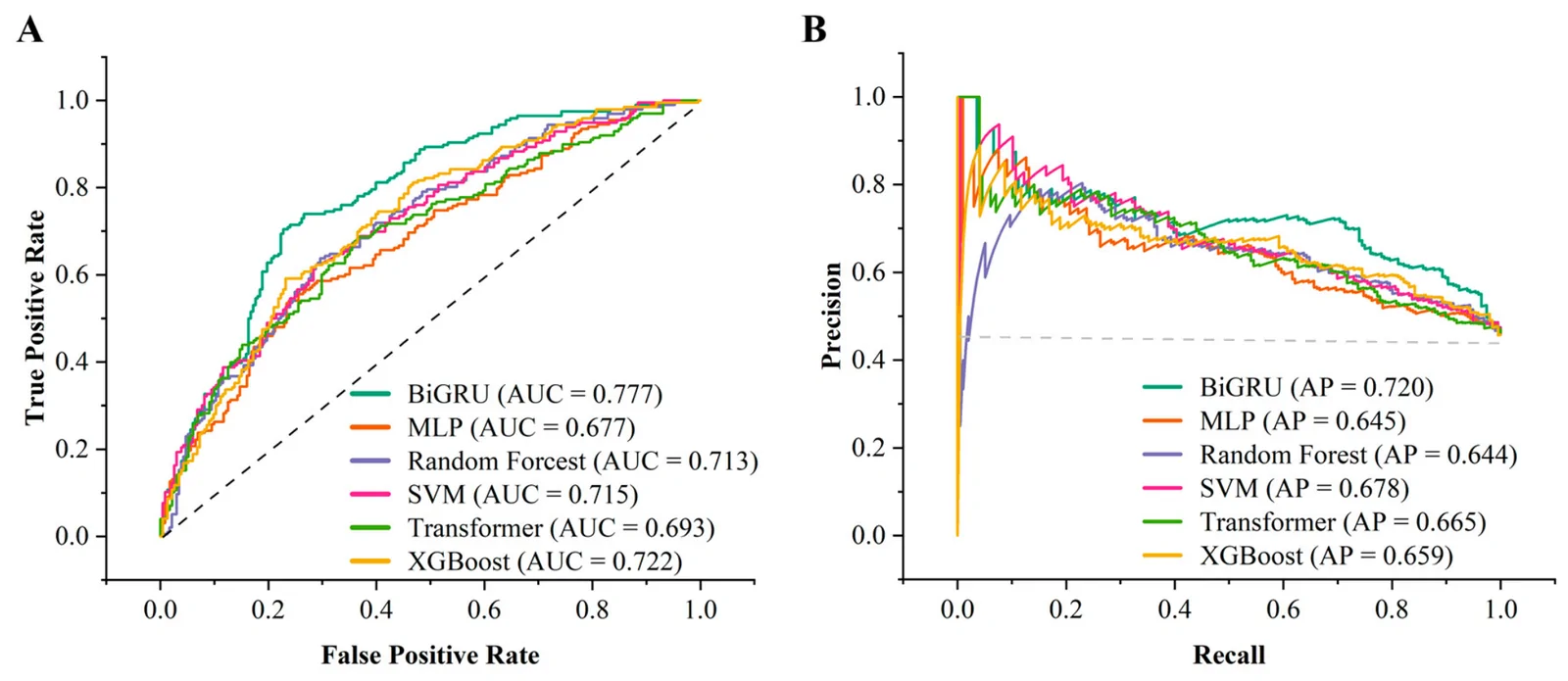
ROC and precision–recall (PR) analysis of six codon expression prediction models. (**A**) ROC curves and corresponding ROC-AUC values. (**B**) PR curves and corresponding average precision (AP) values.

The threshold-independent discrimination ability of the six models was further evaluated using receiver operating characteristic (ROC) and precision–recall (PR) curves (Figure 5). BiGRU achieved the highest ROC-AUC value of 0.777, showing a higher observed ROC-AUC than XGBoost (0.722), SVM (0.715), Random Forest (0.713), Transformer (0.693), and MLP (0.677) (Figure 5A). This result indicates that BiGRU showed the highest observed overall discrimination between high- and low-expression coding sequences across different classification thresholds.

Similarly, BiGRU achieved the highest average precision (AP) value of 0.720 in the PR analysis (Figure 5B). As AP reflects the overall precision–recall performance of a classifier across confidence thresholds, this result further supports the ability of BiGRU to prioritize high-confidence high-expression candidates [41]. Compared with the other models, BiGRU maintained a favorable balance between precision and recall across a broad range of thresholds.

Although some models achieved higher recall, the higher precision and lower false-positive rate of BiGRU were considered more suitable for downstream experimental screening, where only a limited number of candidate sequences could be synthesized and experimentally evaluated. Together with its ROC-AUC and AP results, this performance supported the choice of BiGRU for subsequent coding-sequence ranking.

### 3.4. BiGRU-Guided Codon Optimization and Experimental Validation

Based on the observed predictive performance of BiGRU, the model was subsequently used to prioritize synonymous coding sequences for the selected redesigned lactoferrin variant. Approximately 50% of the codon positions were randomly substituted with synonymous codons while preserving the encoded amino acid sequence. Therefore, the generated coding sequences differed only at the synonymous codon level and encoded the same redesigned lactoferrin protein (Mut-LF). Candidate sequences were scored using the trained BiGRU model, and three high-ranking coding sequences were selected for experimental screening and designated ELF1, ELF2, and ELF3.

**Figure 6:**
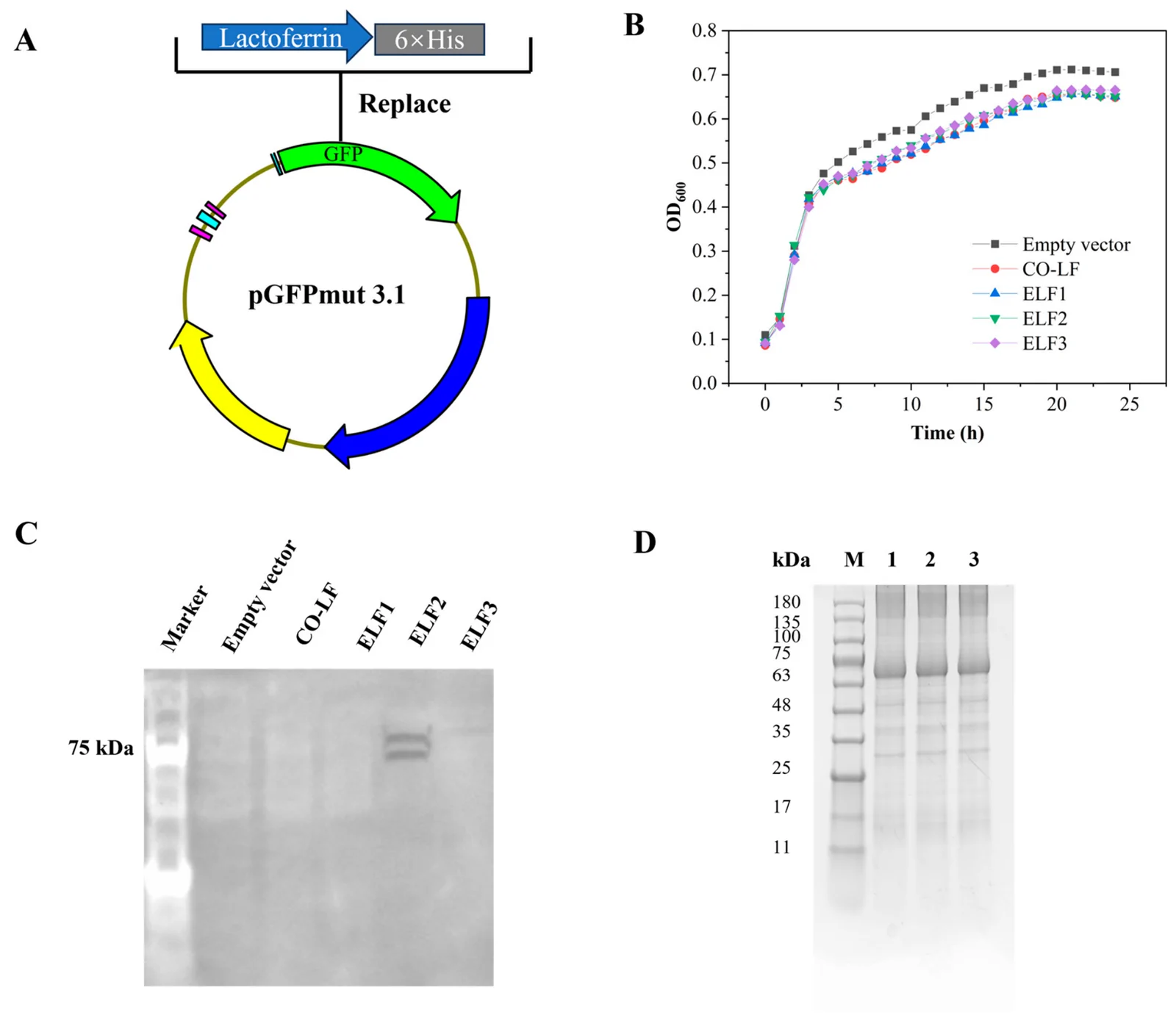
Experimental expression analysis of the WT-LF reference construct and BiGRU-prioritized Mut-LF coding sequences in *Escherichia coli* Nissle 1917 (EcN). (**A**) Schematic of the pGFPmut3.1-based lactoferrin expression constructs carrying a C-terminal 6×His tag. CO-LF encodes WT-LF, whereas ELF1, ELF2, and ELF3 encode the identical Mut-LF amino acid sequence with different synonymous coding sequences. (**B**) Growth curves of EcN carrying the empty vector, CO-LF, ELF1, ELF2, or ELF3 constructs. (**C**) Qualitative Western blot analysis of soluble fractions using an anti-His antibody, with equal amounts of total soluble protein loaded per lane. (**D**) SDS–PAGE analysis of the Ni-NTA eluate obtained from the ELF2-expressing EcN culture; M, molecular-weight marker; lanes 1–3, Ni-NTA eluate samples.

As shown in Figure 6A, the selected coding sequences were synthesized and inserted into the pGFPmut3.1 vector by replacing the GFP coding region. A C-terminal 6×His tag was introduced to facilitate subsequent protein detection. The recombinant constructs, together with the conventionally codon-optimized WT-LF reference construct CO-LF, were transformed into EcN for expression analysis.

Growth curve analysis showed that EcN harboring the CO-LF, ELF1, ELF2, and ELF3 constructs exhibited broadly similar growth profiles during the 24 h cultivation period (Figure 6B). Although slight differences were observed among the strains at later growth stages, no marked growth inhibition was detected in the ELF1–ELF3 groups.

Western blot analysis of soluble fractions loaded with equal amounts of total soluble protein revealed anti-His-reactive signals near the expected molecular-weight region of the encoded product (Figure 6C). For ELF2, two anti-His-reactive bands with different apparent electrophoretic mobilities were observed in the soluble fraction. Among the three BiGRU-prioritized constructs encoding the identical Mut-LF amino acid sequence, a clear anti-His-reactive signal was detected for ELF2, whereas no corresponding bands were detectable for ELF1, ELF3, or the WT-LF reference construct CO-LF under the conditions used. Based on the qualitative soluble-fraction expression profiles, ELF2 was selected for subsequent Ni-NTA enrichment and cell-based evaluation. Following the selection of ELF2, the soluble fraction was subjected to Ni-NTA affinity enrichment. SDS–PAGE analysis of the Ni-NTA eluate showed a predominant protein band near the expected molecular-weight region of the encoded product, together with additional lower-intensity bands (Figure 6D).

The different expression profiles observed for ELF1, ELF2, and ELF3 are notable because all three coding sequences encode the same Mut-LF amino acid sequence. Because these constructs share the same amino acid sequence, their different expression profiles are associated with variation in the synonymous coding sequences under the tested conditions [20]. This observation is consistent with previous studies showing that synonymous coding-sequence composition can influence heterologous protein expression in *E. coli* [42]. Data-driven approaches have also been used for synonymous coding-sequence optimization; for example, Fu et al. developed a BiLSTM-CRF-based codon optimization method and experimentally evaluated the resulting sequences for recombinant protein expression in *E. coli* [43]. CO-LF encodes WT-LF, whereas ELF1–3 encode the redesigned Mut-LF protein, and a conventionally optimized construct encoding Mut-LF was not included. Therefore, the expression profiles of CO-LF and the ELF constructs represent the performance of the respective constructs under the tested conditions, rather than a direct comparison of codon optimization strategies.

The expression results provide a qualitative comparison of anti-His band patterns; soluble yields per culture volume or biomass were not measured in independent cultures. Further characterization using lactoferrin-specific immunodetection or mass spectrometry is needed to identify the anti-His-reactive bands and assess whether the predominant eluate protein is intact Mut-LF.

Taken together, the experimental screening identified ELF2 as a candidate with detectable soluble-fraction anti-His signals among the three model-prioritized sequences tested. ELF2 was therefore selected for Ni-NTA enrichment and subsequent cytokine analysis in NCM460 cells. Further evaluation across a broader range of model-ranked sequences, together with alternative synonymous-sequence designs, would help define the relationship between model scores and experimental expression more systematically.

### 3.5. Cellular Effects of the ELF2-Derived Preparation in NCM460 Cells

**Figure 7:**
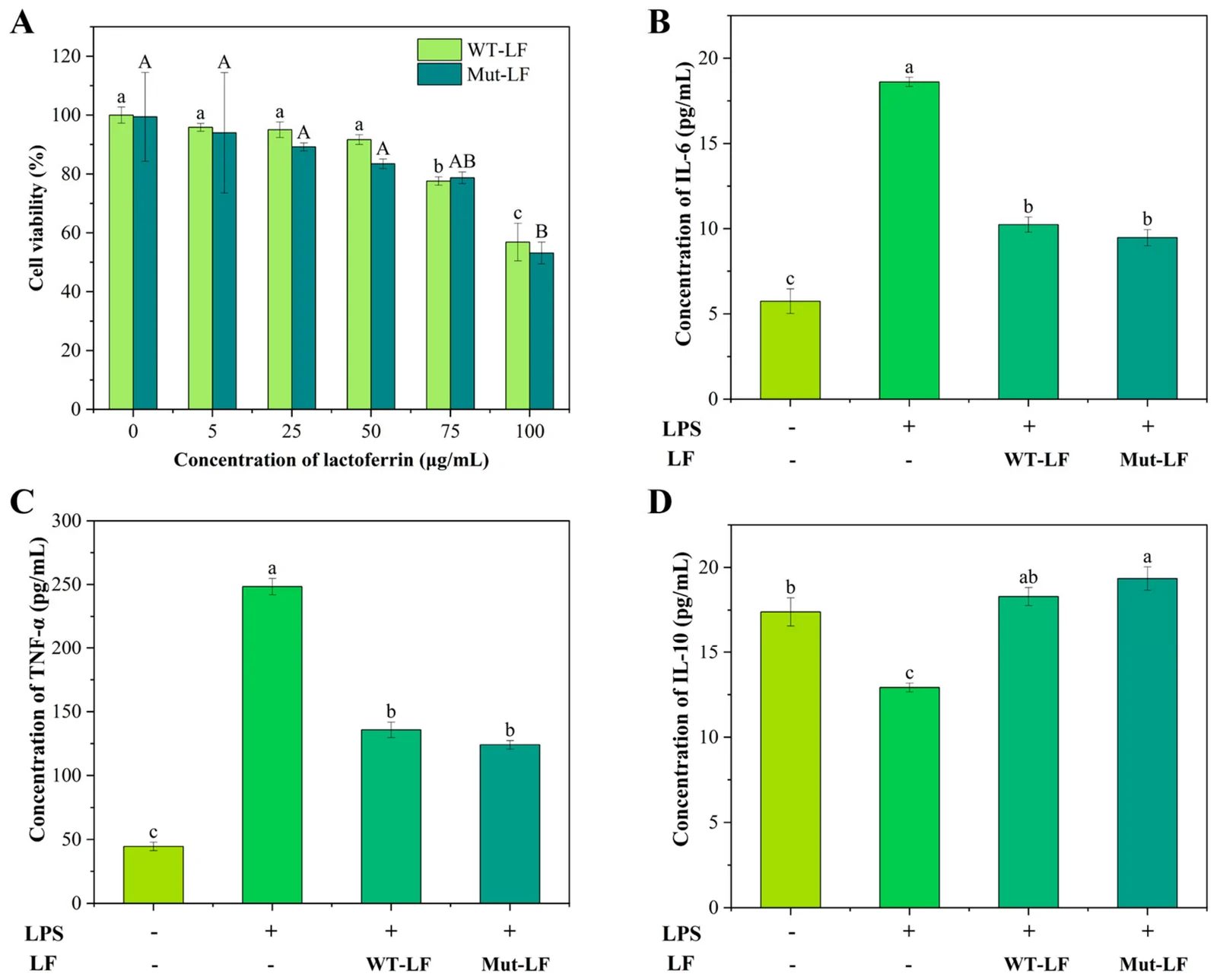
Cellular effects of wild-type lactoferrin (WT-LF) and the ELF2-derived preparation in NCM460 cells. The panel label Mut-LF denotes the ELF2-derived Ni-NTA-enriched preparation. (**A**) Relative CCK-8 signal following treatment with WT-LF or the preparation. (**B**–**D**) IL-6, TNF-α, and IL-10 concentrations (pg/mL), respectively, in culture supernatants from lipopolysaccharide (LPS)-stimulated NCM460 cells following treatment. Data are presented as mean ± SD from three independent biological experiments (*n* = 3). In panel (**A**), different lowercase and uppercase letters indicate significant differences among concentrations within the WT-LF and Mut-LF treatments, respectively; in panels (**B**–**D**), different lowercase letters indicate significant differences among treatment groups (*p* < 0.05).

To evaluate the cellular effects of the ELF2-derived preparation, NCM460 cellular metabolic activity was first assessed using the CCK-8 assay (Figure 7A). At 50 μg/mL, the relative CCK-8 signals were 91.68 ± 1.67% and 83.47 ± 1.62% following treatment with WT-LF and the ELF2-derived preparation, respectively. Based on the concentration-dependent CCK-8 profiles, 50 μg/mL was selected for the subsequent cell experiments.

LPS stimulation markedly increased the levels of the pro-inflammatory cytokines IL-6 and TNF-α (Figure 7B,C). IL-6 increased to 18.61 ± 0.27 pg/mL in the LPS group and decreased to 9.47 ± 0.48 pg/mL after treatment with the ELF2-derived preparation, while TNF-α decreased from 248.40 ± 6.45 pg/mL to 135.81 ± 6.05 pg/mL. Both treatment groups showed significantly lower concentrations than the LPS group, and no significant difference was observed between the two treatment groups. In contrast, LPS reduced IL-10 concentrations, whereas treatment with the ELF2-derived preparation increased IL-10 concentrations (Figure 7D). The preparation increased IL-10 from 12.91 ± 0.26 pg/mL in the LPS group to 18.29 ± 0.53 pg/mL.

Taken together, treatment with the ELF2-derived preparation was associated with lower IL-6 and TNF-α and higher IL-10 concentrations in LPS-stimulated NCM460 cells. Commercial WT-LF served as a functional reference in the cell-based assay, whereas the preparation was obtained from EcN. This pattern is consistent with previous reports showing that recombinant hLF can attenuate LPS-induced inflammatory responses and modulate inflammatory cytokine production [44,45]. Relating these cytokine changes to Mut-LF requires identification and purity assessment of the proteins recovered by Ni-NTA. Endotoxin and co-purifying host material may also have contributed to the cytokine changes. Their effects could not be distinguished in the present assays, as endotoxin was not measured and a mock-purified empty-vector control was not included. The lower CCK-8 signal also requires consideration because cytokines were measured per volume of supernatant. Differences in cell number or metabolism could therefore affect the measured cytokine concentrations. Because CCK-8 was measured in separate wells, it cannot reliably normalize the cytokine measurements retrospectively.

## 4. Conclusions

In this study, an integrated computational–experimental strategy combining protein redesign, structural screening, and BiGRU-guided synonymous codon optimization was developed to support the prioritization of recombinant hLF constructs with favorable soluble-expression profiles in EcN. ProteinMPNN-based redesign followed by SurfSol screening and structural evaluation identified a redesigned lactoferrin variant (Mut-LF) that showed high predicted structural similarity to wild-type lactoferrin, with an RMSD of 0.777 Å and a TM-score of 0.86. Among the six evaluated prediction models, BiGRU showed a performance profile well suited to the precision-oriented screening objective and was subsequently used for coding-sequence prioritization. Qualitative screening of the three model-prioritized candidates detected anti-His-reactive bands in the soluble fraction of ELF2, supporting its prioritization for further characterization. The ELF2-derived Ni-NTA-enriched preparation was associated with lower IL-6 and TNF-α and higher IL-10 concentrations in LPS-stimulated NCM460 cells. Further protein identification and purity assessment are warranted to confirm the presence of Mut-LF and to clarify its contribution to these cellular responses. These findings support the feasibility of integrating protein-level redesign with machine learning-assisted coding-sequence prioritization for the recombinant lactoferrin constructs evaluated in this study.

## Figures and Tables

**Table 1:** Computational screening results for redesigned lactoferrin candidates.

Candidate	SurfSol Score	RMSD (Å)	TM-Score
Mut10	0.36141774	0.865	0.84
Mut31	0.3604095	0.777	0.86
Mut1	0.35614315	NR	NR
Mut5	0.3542963	0.906	0.85
Mut55	0.35029003	1.183	0.86

Note: NR, not reported in the available design data.

## Data Availability

The original contributions presented in this study are included in the article/supplementary material. Further inquiries can be directed to the corresponding authors.

## Supplementary Materials

The following supporting information can be downloaded at: https://www.mdpi.com/article/10.3390/foods15213913/s1, Figure S1: Amino acid sequence alignment of WT-LF and Mut31 (Mut-LF).; Table S1: Primers used for recombinant plasmid construction.
