# Supplementary material for: Machine Learning-Assisted Protein Redesign and Codon Optimization for Soluble Expression of Human Lactoferrin in Escherichia coli
Source: Foods. 2026 Oct 31;15(21):3913. doi: 10.3390/foods15213913 (PMC13762993; doi:10.3390/foods15213913)
Supplement: Supplementary file 1 [file foods-15-03913-s001.zip › Supplementary Figure S1.pdf]

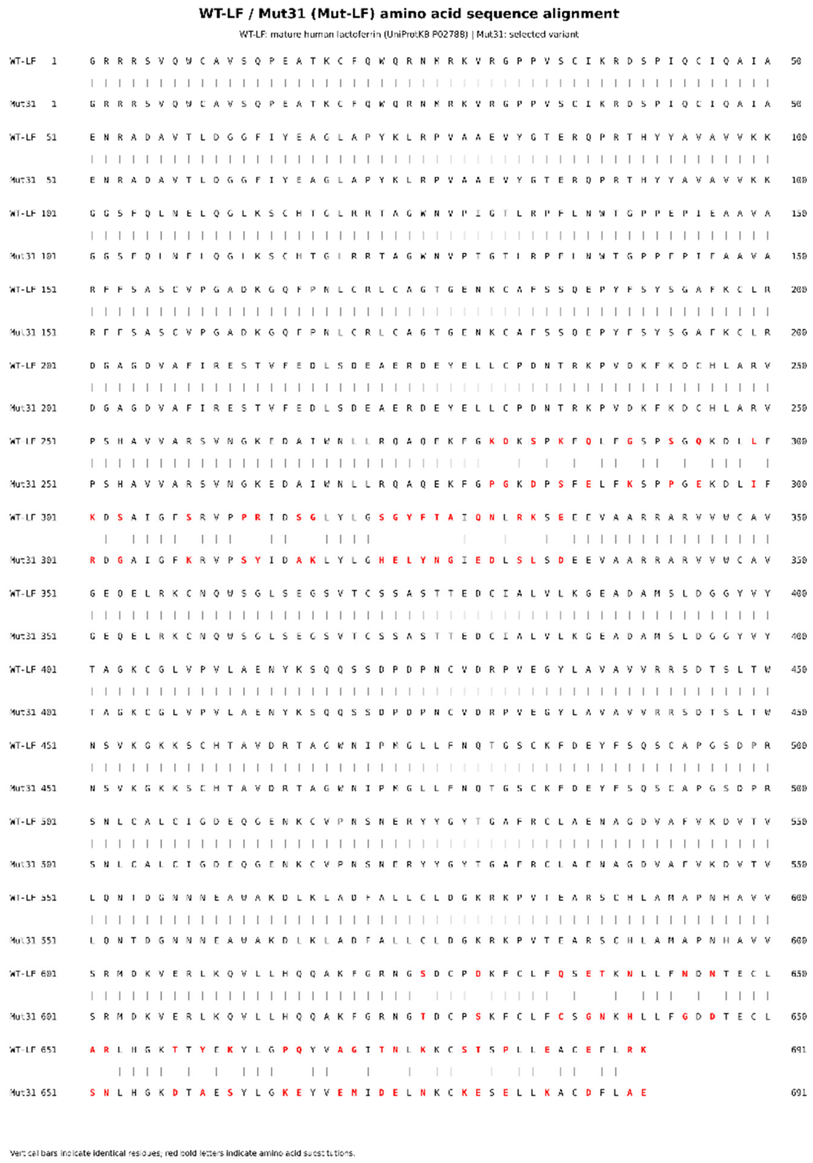

Figure S1. Amino acid sequence alignment of WT-LF and Mut31 (Mut-LF).
