# Supplementary material for: Machine Learning-Assisted Protein Redesign and Codon Optimization for Soluble Expression of Human Lactoferrin in Escherichia coli
Source: Foods. 2026 Oct 31;15(21):3913. doi: 10.3390/foods15213913 (PMC13762993; doi:10.3390/foods15213913)
Supplement: Supplementary file 1 [file foods-15-03913-s001.zip › Supplementary Table S1.pdf]

Table S1. Primers used for recombinant plasmid construction

Primer	Sequence (5′–3′)	Purpose
pmut3.1-F	GCGCAAATAAGCTTAATTAGCTGACCTACTAGTCGG	Vector linearization
pmut3.1-R	CGGCCCATGCTTAATTTCTCCTCTTTAATTCTAGGTACCC	Vector linearization
CO-LF-F	AATTAAGCATGGGCCGCCGTCG	CO-LF amplification
CO-LF-R	CTAATTAAGCTTATTTGCGCAGAAATTCGCACG	CO-LF amplification
ELF1-F	AATTAAGCATGGGCCATGGGTCGTCGTCGTTCTGTT	ELF1 amplification
ELF1-R	CTAATTAAGCTTATTTGCGCCTCTGCTAAGAAGTCGCAGG	ELF1 amplification
ELF2-F	AATTAAGCATGGGCCATGGGTCGTCGTCGTAGCGTG	ELF2 amplification
ELF2-R	CTAATTAAGCTTATTTGCGCTTCTGCTAAAAAGTCACATGCC	ELF2 amplification
ELF3-F	AATTAAGCATGGGCCATGGGACGTCGTCGTTCAGTT	ELF3 amplification
ELF3-R	CTAATTAAGCTTATTTGCGCTTCAGCAAAAATCGCATGCTTT	ELF3 amplification
